# Editorial: Brain dysfunction in Gulf War illness: pathophysiology and treatment

**DOI:** 10.3389/fnins.2025.1642052

**Published:** 2025-06-20

**Authors:** Ashok K. Shetty, Kimberly Sullivan, Liang Qiang

**Affiliations:** ^1^Department of Cell Biology and Genetics, Institute for Regenerative Medicine, Texas A&M University College of Medicine, Bryan/College Station, TX, United States; ^2^Department of Environmental Health, Boston University School of Public Health, Boston, MA, United States; ^3^Department of Neurobiology and Anatomy, Drexel University College of Medicine, Philadelphia, PA, United States

**Keywords:** cognitive and mood impairments, chronic fatigue, deployment-related exposures, Gulf War illness, musculoskeletal pain, neuroinflammation, pesticides

Gulf War Illness (GWI) affects ~30% of the 700,000 veterans who served in the 1990–1991 Gulf War (White et al., 2016). Veterans with GWI display chronic symptoms such as fatigue, headaches, cognitive dysfunction, difficulty concentrating, musculoskeletal pain, and respiratory, gastrointestinal, and dermatologic complaints (White et al., 2016; Janulewicz et al., 2017; Dickey et al., 2021; Krengel et al., 2022). Studies have suggested that multiple deployment-related exposures are common etiologies, including pesticides, chemical warfare nerve gas agents, nerve gas prophylactic medications and other prophylactic treatments, and oil well fire by-products (Steele, 2000; Steele et al., 2012; White et al., 2016; Michalovicz et al., 2020; Steele et al., 2021; Krengel et al., 2024; Keating et al., 2023).

GWI is characterized by persistent cognitive and mood impairments, concentration difficulties, headaches, chronic fatigue, and musculoskeletal pain, which indicate brain dysfunction associated with GWI (White et al., 2016; Janulewicz et al., 2017; Sullivan et al., 2018; Keating et al., 2023). Studies involving veterans with GWI have indicated that cognitive and mood impairments are linked to various adverse changes in neurons, glial cells, and immune cells in the brain (Alshelh et al., 2020; Cheng et al., 2020, 2021). Recent animal model studies have advanced our understanding of the pathophysiology and treatment of brain dysfunction in GWI (Shetty et al., 2020; Madhu et al., 2021; Attaluri et al., 2022; Kodali et al., 2024). These findings may lead to improvements in the quality of life for veterans suffering from GWI. Additionally, clinical trials involving GWI patients have evaluated the effectiveness of various pharmacological and behavioral interventions (Nugent et al., 2021).

This Research Topic collection features eight original research articles and one review article published in Frontiers in Neuroscience, Frontiers in Molecular Neuroscience, Frontiers in Immunology, and Frontiers in Toxicology. These studies, conducted on both veterans with GWI and animal models of GWI, have provided new insights into the disease's pathophysiology and potential treatments. The significant findings from the studies on GW veterans are summarized in the following section.

A study involving 703 Gulf War (GW) veterans assessed their vulnerability to poor health outcomes using a frailty index as a proxy. The findings indicated that, as a group, GW veterans are not frailer than non-GW veterans. However, GW veterans who met the criteria for severe Chronic Multisymptom Illness (CMI) and Kansas GWI were found to be significantly frailer than both other GW veterans and non-GW veterans (Chao). Additionally, the study revealed that GW veterans who met the CMI criteria had higher rates of dementia compared to control GW veterans. Based on these results, the researchers recommended that GW veterans with CMI consider adopting lifestyle changes known to lower the risk of dementia. In another study, Chao et al. evaluated the cognitive status of 952 Gulf War veterans using established neuropsychological criteria and the Montreal Cognitive Assessment (MoCA). They found that 17% of these veterans exhibited mild cognitive impairment (MCI; Chao et al.). Importantly, MCI was found to be linked with CMI, a history of depression, and prolonged exposures related to deployment. A study conducted by Zhang et al. examined 98 veterans who had been deployed during the Gulf War and 90 veterans deployed to Iraq and Afghanistan. The investigation revealed that veterans from both groups had significantly smaller volumes in specific brainstem subregions, along with larger volumes of gray matter in the periaqueductal area (Zhang et al.). Additionally, all veterans showed reduced integrity in the brainstem-spinal cord and brainstem-subcortical tracts. Notably, GWI veterans exhibited structural deficits in the brainstem that were significantly associated with increased sleep difficulties and higher levels of pain. In a study involving 54 Gulf War veterans, Van Riper et al. reported significant increases in strength after 16 weeks of low-to-moderate intensity resistance exercise training. Significantly, this training did not worsen symptoms such as pain, fatigue, or mood (Van Riper et al.). However, the study did not find any correlation between strength, symptoms, and brain structure.

The following section summarizes the new findings from animal models of GWI within this research collection. Carpenter et al. examined the progression of structural changes over 12 months in two mouse models: the pyridostigmine bromide (PB) and permethrin (PER) model, and the PB, N,N-diethyl-meta-toluamide (DEET,) corticosterone (CORT) and Diisopropyl fluorophosphate (DFP) model (Carpenter et al.). The study reported that both models exhibited ventricular enlargement and reductions in hippocampal volumes as they aged. Additionally, the PB/DEET/CORT/DFP model showed decreased brainstem and total brain volumes, while the PB/PER model experienced reduced cortical thickness (Carpenter et al.). In another study, Mozhui et al. used a mouse model exposed to CORT and DFP, reporting the differential expression of 67 methylated genes associated with various symptoms of GWI. This finding suggests that GWI may be linked to significant epigenetic changes (Mozhui et al.). Additionally, research conducted by Shaikh et al. involved a mouse model exposed to PB, chlorpyrifos, and DEET, demonstrating that an Ayurvedic Withania somnifera root extract treatment could offer neuroprotection, particularly by preventing the loss of dendritic spines on neurons (Shaikh et al.). Furthermore, an investigation by Terry et al. revealed that acute exposure to DFP not only results in persistent cognitive impairments but also increases signs of cellular senescence in the brain (Terry et al.).

In addition to the original research articles discussed above, the Research Topic collection includes a review article that describes the latent phenotype of GWI. The review summarized a possible link between the dysregulated of immune and endocrine signaling and progressive cognitive impairments in GWI (Burzynski and Reagan). The dysregulated immune and endocrine signaling comprised chronic neuroinflammation, as well as disruptions in central cholinergic signaling, which are particularly seen in the presence of stressors. Additional perspectives in the review include the implication of repeated activation of a sensitized cholinergic system. It is proposed that dysregulated acetylcholine signaling can lead to the potentiation of peripheral and central inflammation, as well as accelerate cognitive decline (Burzynski and Reagan). Such possibilities are supported by clinical data demonstrating exacerbation of GWI-related cognitive impairments when GWI patients undergo an exercise challenge (Burzynski and Reagan).

In summary, the article collection in this Research Topic provides several novel insights on the pathophysiology of GWI.
